# Endoscopic Management of Bleeding in Altered Anatomy after Upper Gastrointestinal Surgery

**DOI:** 10.3390/medicina59111941

**Published:** 2023-11-02

**Authors:** Giulia Gibiino, Cecilia Binda, Matteo Secco, Paolo Giuffrida, Chiara Coluccio, Barbara Perini, Stefano Fabbri, Elisa Liverani, Carlo Felix Maria Jung, Carlo Fabbri

**Affiliations:** 1 Gastroenterology and Digestive Endoscopy Unit Ospedale Morgagni—Pierantoni, Forlì—Ospedale M. Bufalini, Cesena—AUSL, 47521 Romagna, Italy; giulia.gibiino@gmail.com (G.G.); matteo.secco01@gmail.com (M.S.); paologiuffrida1@gmail.com (P.G.); colucciochiara@gmail.com (C.C.); barbara.perini@studenti.unipd.it (B.P.); stefano.fabbri20@gmail.com (S.F.); elisa.liverani@auslromagna.it (E.L.); carlo.jung@gmx.de (C.F.M.J.); carlo.fabbri@auslromagna.it (C.F.); 2 Department of Surgery, Oncology and Gastroenterology (DISCOG), University Hospital of Padua, 35128 Padua, Italy

**Keywords:** non-variceal upper gastrointestinal haemorrhage (NVUGIH), bariatric surgery, altered anatomy, anastomotic bleeding, marginal ulcers, endoscopic therapy

## Abstract

Postoperative non variceal upper gastrointestinal haemorrhage may occur early or late and affect a variable percentage of patients—up to about 2%. Most cases of intraluminal bleeding are an indication for urgent Esophagogastroduodenoscopy (EGD) and require endoscopic haemostatic treatment. In addition to the approach usually adopted in non-variceal upper haemorrhages, these cases may be burdened with difficulties in terms of anastomotic tissue, angled positions, and the risk of further complications. There is also extreme variability related to the type of surgery performed, in the context of oncological disease or bariatric surgery. At the same time, the world of haemostatic devices available in digestive endoscopy is increasing, meeting high efficacy rates and attempting to treat even the most complex cases. Our narrative review summarises the current evidence in terms of different approaches to endoscopic haemostasis in upper bleeding in altered anatomy after surgery, proposing an up-to-date guidance for endoscopic clinicians and at the same time, highlighting areas of future scientific research.

## 1. Introduction and Epidemiology

A wide range of surgical procedures altering the anatomy of the upper gastrointestinal tract is performed every year worldwide. Most surgical procedures performed on the upper gastrointestinal tract can be ascribed to intended oncologic surgery or bariatric metabolic surgery (BMS). The number of BMS procedures completed continues to grow, with more than 685,000 procedures having been performed worldwide in 2016 alone [1], and the same concept applies to oncologic surgery. This continuous and unrelenting increase in the number of surgical minimally invasive procedures aligns with the increasing number of postoperative complications that can occur, like anastomotic haemorrhage, anastomotic insufficiency, delayed gastric emptying and anastomotic stenosis [2,3]. These problems can be successfully managed by surgeons, interventional radiologists, or endoscopists. In the forementioned complications, above all, for endoluminal bleeding, endoscopy plays a central role in its diagnosis and subsequent treatment [3,4,5]. Moreover, postoperative bleeding can prolong hospital stay and result in morbidity and mortality, particularly in critically ill patients [6]. Postoperative bleeding can be extraluminal, which is mostly linked to bleeding from abdominal vessels and requires radiological or surgical management, or even intraluminal management, in which endoscopy plays a crucial role.

Intraluminal haemorrhage occurs mostly in the early period after surgical intervention, as reported by Shen Y. et al. and Jamil et al. [7,8]. Different surgical procedures can be performed on the upper GI tract, leading to wide ranges of incidence of post procedural bleeding. In the literature, the rate of bleeding following gastrectomy or esophagectomy for oesophageal cancer has been estimated to be as high as 2% of patients undergoing surgery [9].

In several case series of patients undergoing surgery for gastric cancer, postoperative bleeding occurred in 0.3% to 2.7% of patients [3,5]. In two case series evaluating bleeding after gastric bypass surgery, the incidence of intraluminal bleeding was 0.49% and 3.2%, respectively [7,10]. When considering pancreaticoduodenectomy, the incidence of postoperative intraluminal GI bleeding in the literature is higher and is estimated to be from 2.2% to 8.4% [11,12,13]. These high incidence rates might be justified by the complexity of the procedure itself and by the involvement of three anastomoses (gastrojejunostomy, hepaticojejunostomy, and pancreaticojejunostomy). The timing of bleeding should also be taken into consideration, as it may be related to different underlying causes. Indeed, early bleeding usually occurs at the staple lines of the anastomosis, while delayed bleeding is usually secondary to an anastomotic ulcer [14]. In a retrospective study from Song W. et al. including 1536 patients who underwent surgery for gastric cancer with subsequent gastrectomy, 1.2% of patients developed early postoperative haemorrhage (defined as bleeding emerging within the first 24 h after surgery), while 0.9% experienced delayed massive haemorrhage (defined as bleeding emerging after the first 24 h after surgery) [15]. Even so, defining the exact incidence of the problem may be complicated because of different definitions for early and delayed bleeding after surgery throughout the studies.

The aim of our review is to focus on the endoscopic management of upper gastrointestinal bleeding in patients with surgically altered anatomy.

## 2. Materials and Methods

We selected articles discussing the topic of bleeding in altered anatomy after surgery in the upper gastrointestinal tract, paying specific attention to magnitude of the problem and the endoscopic management. We developed a non-systematic review article using the following electronic sources: PubMed, EMBASE, Google Scholar, Ovid, MEDLINE, Scopus, the Cochrane controlled trials register, and Web of Science. We used the following search terms alone and in combination: “non variceal upper gastrointestinal hemorrhage”, “NVUGIH”, “endoscopic treatment”, “marginal ulcers”, “gastric bleeding”, “surgery”, “altered anatomy”, “Roux-en y gastric bypass”, “bleeding”, “hemorrhage”, and “gastrectomy”. We examined all the articles reporting data related to humans (inclusion criterion) while excluding works with no full text available, works that were not in the English language, book chapters and abstracts, articles published before 1990, and articles not concerning surgically altered anatomy (exclusion criteria). We did not consider a minimum number of cases for each paper, considering that the available literature consists mainly of case reports and case series. Finally, we evaluated supplementary references among the articles evaluated in the first search round.

In Table 1 we summarized the papers taken in account for this review and their results.

## 3. Diagnosis

Postoperative bleeding is usually diagnosed with signs of bleeding associated with a drop of haemoglobin of more than 2 g/dL. Bleeding can be further categorized as intrabdominal bleeding, which is mostly linked to arterial bleeding from abdominal vessels (with a radiological or surgical management), and intraluminal bleeding, which can be managed with endoscopic treatment.

For intraluminal bleeding, the most frequent clinical presentation is hematemesis, followed by melena/haematochezia or bleeding through a nasogastric tube [21].

Postoperative gastrointestinal bleeding can be caused by surgery or depend on conditions that are related to surgery. One of the most common causes of intraluminal haemorrhage is represented by marginal ulcers, which are the consequence of mucosal ischemia originating from perfusion defects, anastomosis tension, or sutures material [23]. Another important cause of bleeding after surgery is linked to stress-related ulcers, which can develop in the upper GI as a consequence of the stress produced by the surgical intervention. Mallory-Weiss tears, resulting from nausea and repeated vomiting can be a source of bleeding related to surgery [24].

A particular scenario that should be kept in mind is bleeding from the gastric remnant after Roux-en-Y gastric bypass (RYGB). In this condition, the bleeding source is located in the bypassed stomach, making endoscopic treatment challenging since the source of bleeding is barely reachable with standard techniques. Double-balloon enteroscopy has diagnostic and therapeutic value for these patients since it allows the endoscopist to reach the bleeding site and perform endoscopic treatment, however its availability and the skills required represent a huge limitation of these technique. These limitations make the surgical approach the standard of care for most of these patients.

After volume resuscitation with fluids and blood products and achieving the target haemoglobin level of 7 mg/dL (9 mg/dL for patients with significant cardiovascular diseases), endoscopic evaluation for assessing the source of bleeding and treatment is the subsequent step.

To date, there is still no specific indication about the best timing for endoscopic evaluation in the context of intraluminal bleeding after surgery. In the past, there was concern about the possibility of anastomotic damage and perforation during endoscopic procedures performed immediately after surgical intervention This concern is now no longer relevant since endoscopy was reported to be safe without significant complications, even in the early postoperative period [7,18,25].

Furthermore, in case of bleeding involving the extraluminal component, endoscopy can fail to detect haemorrhages, delaying the appropriate intervention, such as angiographic embolization or surgery [26]. We will refer then to the timing established by ESGE guidelines for the management of non-variceal upper gastrointestinal haemorrhage, which suggest early endoscopy (<24 h from patient presentation) since it is associated with lower in-hospital mortality, shorter lengths of stay, and lower total hospital costs [27].

**Figure 1:**
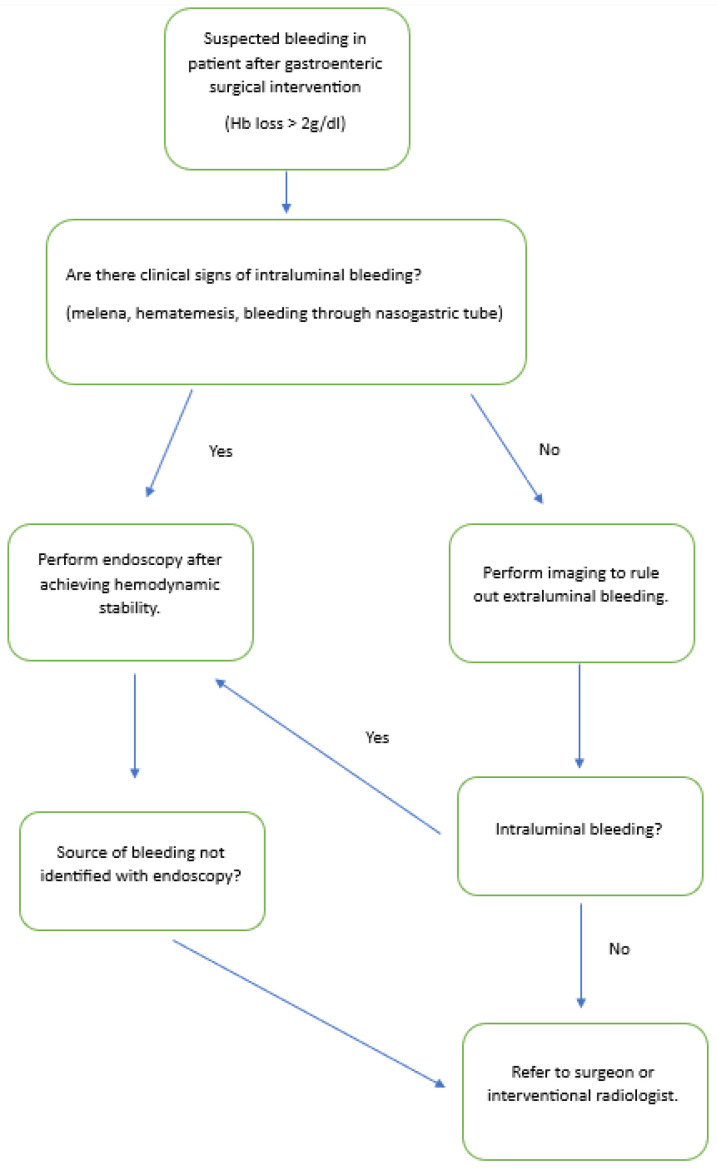
Flowchart for the diagnostic management of bleeding in patients with surgically altered anatomy.

We propose hereafter a flowchart for the diagnostic management of bleeding in patients with surgically altered anatomy (Figure 1).

The success rate of endoscopic haemostasis is strongly related to the type of surgery performed and, therefore, to the varying degree of accessibility to the bleeding focus. The endoscopic approach is simpler in patients who underwent gastrectomy or gastrojejunostomy compared to patients who underwent pancreaticoduodenectomy or Roux-en-Y gastric bypass [5,28].

The lesions found are usually classified in daily practice according to the Forrest classification, usually adopted for peptic ulcers and related to the risk of rebleeding [29]. However, in the case of marginal ulcers, Shin first proposed a classification in 1994 considering active bleeding (spurting or oozing), visible vessel, and blood clot adhesion [30]. The variety of lesions encountered and the experience of the centre contribute to the lack of homogeneity in the descriptions provided and to the lack of a single recommended classification in the literature to be adopted in these circumstances.

## 4. Endoscopic Haemostasis

The endoscopic treatment of haemorrhage in the gastrointestinal tract is based on different procedures and is sometimes combined with injection therapy, mechanical approach, thermal haemostasis, the application of absorbing substances, or other less common approaches. All these options are also available for anastomotic haemorrhage. The choice of haemostasis technique, especially in the context of anastomotic haemorrhage, depends mostly on the preference of the endoscopist and the time passed from the creation of the anastomosis. Clinical papers exclusively reporting on the outcomes on haemostasis techniques in the context of altered anatomy after surgery are rare and no randomized controlled trials has been conducted to compare the efficacy of different modalities for treating postoperative haemorrhage. Experience is often derived from larger case histories of haemostasis where some cases of bleeding in the upper digestive tract surgery outcomes are included. In addition to the problem of first bleeding in these cases, any rebleeding cases may also be complex and require infrequent procedures. In the subsequent section we will briefly summarize the methods of haemostasis available so as to guide clinical activity on the basis of the literature published to date.

### 4.1. Injection Therapy

Injection is based on local tamponade resulting from the volume effect in the submucosal layer. It can be achieved using diluted epinephrine (1:10,000 or 1:20,000). This allows for pressure elevation and also a secondary effect of vasoconstriction in the same submucosal layer. Alternative injection can be performed by using sclerosing agents or tissue adhesives, including cyanoacrylate glues commonly used to create a primary clog at the bleeding site.

The technique usually requires needles consisting of an outer sheath and an inner hollow-core needle (19–25 gauge). The injection is usually performed near the bleeding site. Clearly in surgical outcomes it may be difficult to maintain a stable position or obtain effective access to the submucosal layer.

The advantage of this technique is the wide availability and the low costs, the biggest limitation is the lability of the haemostasis obtained, resulting in the need for the application of a second haemostasis technique [31].

### 4.2. Mechanical Haemostasis

Endoscopic mechanical therapies include clips (through-the-scope (TTS) and over-the-scope-clips (OTSCs) or cap-mounted) and band ligation devices. The latter are usually employed for oesophageal variceal bleeding but have also been reported for the treatment of NVUGIH (e.g., Dieulafoy lesions). The mechanism is rather simple and involves the placement of elastic bands over tissue to produce mechanical compression and tamponade [27]. As far as clips on the market are concerned, there is considerable variety in terms of size, manoeuvrability, and repositioning options.

#### 4.2.1. Through-The-Scope-Clips

The endoclip or haemoclip is a mechanical method of haemostasis introduced in 1975. This is the most widely applied method because of its simplicity, reduced costs, wide availability, and low risk of adverse events in the treatment of non-variceal upper gastrointestinal haemorrhage [32]. Through-the-scope-clips can be applied through the working channel, allowing the operator to maintain a clear view over the bleeding source. Haemoclips can be safely used for anastomotic bleeding since they have the advantage of not injuring the surrounding tissue [18].

However, haemoclips application can be technically difficult due to a poor axis caused by anatomical distortion after surgery. Furthermore, there is no evidence supporting the reduction in terms of rebleeding in this specific setting. Lee et al. [20]. Reviewed and discussed the efficacy of endoscopic haemoclipping and dual therapy with epinephrine injection and heater probe thermocoagulation with respect to the haemostatic rates, recurrent bleeding rates, transfusion requirements, the need for surgery, and mortality rates in bleeding marginal ulcers after partial gastrectomy. Among fifty cases, thirty patients underwent dual therapy and the remaining twenty patients received haemoclipping depending on endoscopist’s expertise or preference. The initial endoscopic haemostasis rate was 100% in both groups, but the rebleeding rate within 30 days was significantly different in the two groups in favour of haemoclip treatment (5% in the haemoclipping group versus 33% in the thermocoagulation one). Further results included a low rate for the need for surgery (4%) and hospital mortality (4%) with no procedure-related consequences observed.

#### 4.2.2. Over-The-Scope-Clips (OTSCs)

OTSCs are a great tool for the primary treatment of bleeding and retreatment after failure or recurrence of haemorrhage. OTSC use, since its first description in 2007, has become one of the standard procedures for bleeding in the gastrointestinal tract and has showed increasing efficacy in treatment and retreatment of haemorrhage in the upper gastrointestinal tract [33].

In 2017, Honegger et al. reported a large OTSC cohort placement including cases of bleeding occurring in iatrogenic circumstances or in gastric bypass [34]. Their experience has shown success rates of up to 90% in these cases, showing the importance of these devices in daily endoscopic practice. The most recent STING-2 trial showed the role of primary OTSC therapy, which is superior to standard treatment with clips, for selected cases of non-variceal upper gastrointestinal bleeding (NVUGIB) with a high risk of rebleeding [33]. Out of 246 total patients, a small percentage corresponded to bleeding from an anastomotic origin—3 (5.8%) to standard treatment and 3 (6.2%), respectively. Considering the 30-day outcomes, only one case from the latter group underwent surgical revision for persistent anaemia, showing OTSC in situ and substantial long-term technical success. Tontini et al. [22] published a case report of anastomotic bleeding occurring in a 71-year-old woman, previously submitted to laparotomic debridement with a Billroth I gastroenteric anastomosis for an advanced ovarian tumour with massive peritoneal carcinomatosis. Active haemorrhage was attributed to oozing bleeding from an anastomotic dehiscence at the posterior wall. The haemostasis was achieved by injection therapy combined with “atraumatic” OTSCs. This is an example of a successful application in relation to the angled position, large leak, and massive bleeding. OTSCs grant a high closure power, so an impairment of anastomotic blood circulation due to the OTSCs could be possible. Beyond these specific cases, however, there are no case series or RCTs on the use of OTSCs in bleeding from surgical anastomosis able to provide evidence supporting their use as a primary device.

### 4.3. Thermal Haemostasis

Contact probes have been available for thermal haemostasis since the 1970s. Contact probes can provide haemostasis with two different mechanisms: pressure on the blood vessel, thereby stopping the blood flow, and thermal energy cauterizing the vessel underneath by coaptive coagulation. Possible contact probes of the thermic haemostasis techniques are the dry monopolar electrode, the liquid electrode, the bipolar electrode, the heater probe, the Coagrasper haemostatic forceps, or hot biopsy forceps. Thermal probes can be furtherly divided into contact devices, for which contact with the site to treat is required, and noncontact devices, for which touching the target tissue is not necessary. The main risk of using a thermal probe is perforation linked to the excessive application of coagulation or pressure, especially in acute or non-fibrotic lesions. Thermal probes can also cause a coagulation injury that can make larger and deeper lesions, and may increase the risk of delayed bleeding, particularly in patients with a coagulopathy [35].

Argon plasma coagulation (APC) is a noncontact device because the current follows the path of the lowest tissue resistance without necessarily touching the tissue. It uses a high-frequency, monopolar alternating current that is conducted to the target tissue without mechanical contact, resulting in the coagulation of superficial tissue. Disadvantages and advantages of APC are similar to those of contact probes [36].

The study conducted by Lee in 2002 [20] is the only one to provide a comparison of combined injective and thermal haemostasis versus haemoclips with considerable risk of rebleeding, as outlined above. In view of the risks associated with this type of haemostasis, it is a modality that is often not used in the first instance in operated stomachs as well as in the case of recent oesophagogastrostomies or jejunostomies. A Special Issue should be considered in case of bleeding from stapled anastomosis due to the increased risk of bleeding [37]. The use of thermal modalities of haemostasis in this setting is burdened by a risk, albeit low, of major complications like ischemic injury and perforation. Its use on frail tissue, like staple line in the context of altered anatomy, should be restricted to those cases where other endoscopic modalities do not achieve bleeding control or cannot be performed for anatomic reasons. However, bleeding from stapled anastomosis is more frequently observed intraoperatively or managed by surgeons.

### 4.4. Topical Agents

Haemostatic powders (HPs) and haemostatic gels act as “touch-free” agents that can be used for the treatment of gastrointestinal bleeding and are generally safe and well tolerated [38]. The advantages of noncontact spray-catheter delivery of haemostatic agents include the ease of use, efficacy even in absence of precise lesion targeting, access to lesions in angulated positions, the possibility of use in combination with other haemostatic modalities, and the ability to treat a larger surface area. On the other hand, they are not always available in everyday endoscopic practice and can be expensive. The topical agents currently available in Western countries include: Hemospray, also known as TC-325 (HS, Cook Medical, Winston-Salem, NC, USA); EndoClot (EC, Micro-Tech Europe, Düsseldorf, Germany); and PuraStat (3-D Matrix Europe SAS, Caluire-et-Cuire, France).

Haemostatic powder application does not require accurate spraying at bleeding sites, which are frequently difficult to visualize in an endoscopic view and to approach. The most important disadvantage is the possibility of clogging of the application catheter, which may occur when the powder is in contact with the fluid inside the catheter. Additionally, the visibility of the bleeding site and landmarks can become obscured following the application of the powders owing to their opaque nature [39].

Hemospray is an inert mineral based compound, which, in contact with fluids, absorbs water and acts cohesively forming a covering mechanical tamponade. Through fluid absorption, it favours clot formation by deforming and packing erythrocytes, it also concentrates activated platelets with clotting factors and interacts with the fibrin matrix, and within 24 to 72 h, the adherent coat sloughs off into the GI lumen [40,41]. Because of these local haemostatic proprieties, first studies suggest that HS is equally effective in both patients with and without systemic antithrombotic therapy [42]. The fact of being equally effective in patients with or without antithrombotic therapy could be of great importance in the context of bleeding after bariatric surgery since most patients receive thromboembolic prophylaxis in the perioperative period followed by twice-daily subcutaneous injections of low-molecular-weight heparin until discharge [7].A single case report by Granata et al. showed efficacy and safety in the treatment of bleeding from gastroenteric anastomosis performed for a duodenal bulb stenosis [19].

Another feature that could be important in treatment of bleeding from anastomosis is that HS is sprayed at high pressure with a propellant CO_2_ cartridge. Such a feature can potentially cause further tissue injury to the point of perforation especially in friable or inflamed mucosa, as reported by Vitali F. et al. in two patients treated with HS [38]. In cases of diffuse bleeding in which haemostasis is not achieved by conventional modalities, Hemospray can be used in the early postoperative period [43].

Endoclot is a powder agent composed of absorbable haemostatic polysaccharides. When in contact with blood, EC initiates a dehydration process promoting the concentration of clotting factors, platelet, and erythrocytes, thereby accelerating the clotting process and the formation of a mechanical shell of gelled matrix, which adheres to the bleeding tissue [44]. Data on the efficacy of EC suggest that EC allows for excellent bleeding control when applied as primary or salvage therapy. Furthermore, the EC pressure when spraying is much lower than that of HS, allowing for a more sectorial area of targeting, making EC more suitable for localized bleeding lesions, like peptic ulcers or a surface after resection, reducing the low, albeit reported, risk of perforation [38].

Haemostatic gels scaffold the self-assembling peptide technology and are indicative of the haemostasis of oozing bleeding in parenchyma of solid organs, vascular anastomoses, and small blood vessels or capillaries of the GI.

PuraStat is comprised of a repeating sequence of amino acid, Arginine^®^, Alanine (A), and Aspartic Acid (D). Once the PuraStat gel is in direct contact with blood or bodily fluids, the acidic peptide solution is rapidly released, resulting in the ß-sheets forming a three-dimensional hydrogold nano-fibre that is similar in structure to the extracellular matrix. This hydrogel matrix forms a physical barrier over the bleeding vessel or area to cause haemostasis. It is applied via a dedicated endoscopic catheter through the working channel of any diagnostic or therapeutic endoscope.

It has proven effectiveness in reducing delayed bleeding following tract endoscopic submucosal dissection (ESD), [45,46,47,48]. There are no studies demonstrating efficacy in high digestive bleeding in surgical outcomes, but it is expected that it may prove useful in these cases due to the visibility of the bleeding area remaining intact, and the endoscope view not being obscured due to the transparent nature of the peptide; therefore, haemostasis is under the direct visual control of the physician. In addition, due to the hydrogel nature of the device, the catheter does not become clogged by the product [39].

### 4.5. Suturing and Stenting

Endoscopic suturing and stenting have been introduced as rescue therapies prior to revisional surgery for marginal ulcer bleeding not responding to conventional endoscopic therapy [49].

A case report was published by Barola et al. in 2017 [17] showing endoscopic suturing as a novel technique to treat massively bleeding marginal ulceration post RYGB. They described this approach as technically safe, feasible, and a possible life-saving technique to be considered as an alternative to surgery [50]. Subsequently, the same group of authors successfully reported a case series of 11 patients also suffering from MU post-Roux-en-Y gastric bypass (RYGB) outcomes treated with suturing devices and/or fully covered self-expandable metallic stents (FCSEMS). This is the first manuscript considering this indication for FCSEMS in order to cover the ulcer bed to treat MUs in case the gastric outlet is of an insufficient calibre to allow for endoscopic oversewing [16].

Although these are mainly anecdotal experiences of very experienced centres, an increase in these possible approaches is to be expected given the growing number of bariatric surgery outcomes.

## 5. Conclusions

**Figure 2:**
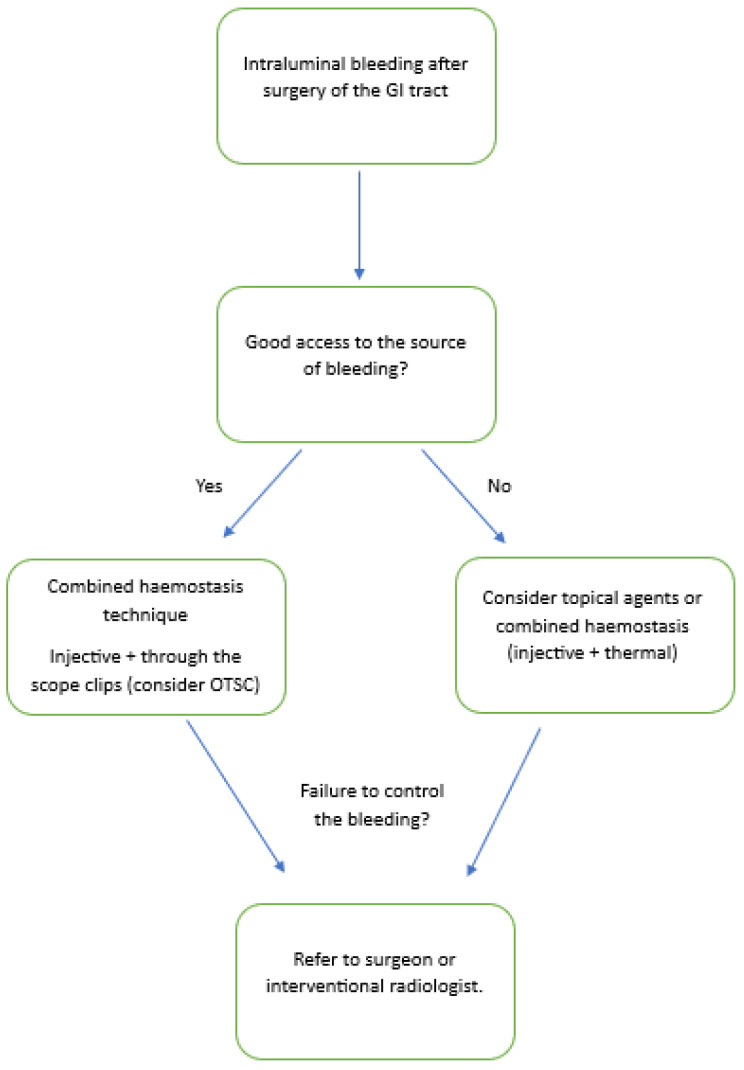
Proposed flowchart showing the possible endoscopic approach in the treatment of bleeding.

Gastric bleeding occurring in altered anatomy after surgery is not uncommon and can be a challenging condition even in the hands of experienced endoscopists [5]. The presentation of intraluminal bleeding usually requires an early endoscopic evaluation, although to date, data are lacking regarding the best timing. The haemostasis and endoscopic approach do not differ from the usual techniques adopted in NVUGIH. However, difficulties may be greater depending on the location, tissue type, and consistency and effectiveness of the various methods adopted [15]. The current literature has demonstrated the effectiveness of using mechanical haemostasis with clips, such as haemoclip or OTSCs, both immediately and in terms of rebleeding, which probably should be considered as the first haemostatic approach in this setting [33]. At the same time, several topical agents that are currently on the market and increasingly on the rise may be the easiest devices to use in this setting. The growing number of people being operated for oncological reasons or obesity will increasingly place us in front of this need, and new devices will have to be tested. Even considering the limitations linked to the absence of randomized controlled trials comparing endoscopic management options in intraluminal bleeding after surgery, we tried to propose a simple flowchart showing the alternatives available for the treatment of bleeding (Figure 2). Large prospective studies and randomized clinical trials are needed to define the best endoscopic approach in these patients, which are often fragile, coming after respective surgery.

## Figures and Tables

**Table 1:** Summary of the papers taken in account for this review and their results.

Author	Publication Year	Study	N° of Patients Included	Surgery	N° of Patients with Bleeding	N° Patients with Endoscopic Treatment of Bleeding
Barola S. [16]	2018	Case series	11	RYGB	11	11
Barola S. [17]	2017	Case report	1	RYGB	1	1
Heneghan M. H. [10]	2011	Case series	4466	RYGB	42	0
Wichmann D. [9]	2022	Review	758	-	-	-
Tang S. J. [18]	2007	Case report	2	RYGB	2	2
Song W. [15]	2014	Case series	1534	gastrectomy	33	7
Shen Y. [8]	2023	Case series	2978	gastrectomy	85	3
Granata A. [19]	2015	Case report	1	gastroenteric anastomosis	1	1
Lym S. G. [3]	2012	Review	393	gastrectomy	9	9
Jamil H. L. [7]	2008	Case series	933	RYGB	30	30
Kim K-H. [5]	2012	Case series	2031	gastrectomy	7	7
Lee Y-C. [20]	2002	Comparative study	50	subtotal gastrectomy	50	50
Park J.Y. [21]	2014	Case series	5739	gastrectomy	48	2
Tontini G. E. [22]	2013	Case report	1	subtotal gastrectomy	1	1

## Data Availability

Not applicable.

## References

[1] Angrisani L., Santonicola A., Iovino P., Vitiello A., Higa K., Himpens J., Buchwald H., Scopinaro N. (2018). IFSO Worldwide Survey 2016: Primary, Endoluminal, and Revisional Procedures. Obes. Surg..

[2] Livingston E.H. (2004). Procedure incidence and in-hospital complication rates of bariatric surgery in the United States. Am. J. Surg..

[3] Lim S.G., Lee K.M., Kim S.S., Kim J.S., Hwang J.C., Shin S.J., Han S.-U., Kim J.H., Cho S.W. (2012). Endoscopic Approach for Postoperative Complications Following Laparoscopic-Assisted Gastrectomy in Early Gastric Cancer: Literature Review. Hepatogastroenterology.

[4] Park J.-H., Jeong S.-H., Lee Y.-J., Kim T.H., Kim J.-M., Kim D.-H., Kwag S.-J., Kim J.-Y., Park T., Jeong C.-Y. (2020). Safety and efficacy of post-anastomotic intraoperative endoscopy to avoid early anastomotic complications during gastrectomy for gastric cancer. Surg. Endosc..

[5] Kim K.-H., Kim M.-C., Jung G.-J., Jang J.-S., Choi S.-R. (2012). Endoscopic treatment and risk factors of postoperative anastomotic bleeding after gastrectomy for gastric cancer. Int. J. Surg..

[6] Jeong O., Park Y.K., Ryu S.Y., Kim D.Y., Kim H.K., Jeong M.R. (2011). Predisposing factors and management of postoperative bleeding after radical gastrectomy for gastric carcinoma. Surg. Today.

[7] Jamil L.H., Krause K.R., Chengelis D.L., Jury R.P., Jackson C.M., Cannon M.E., Duffy M.C. (2008). Endoscopic management of early upper gastrointestinal hemorrhage following laparoscopic Roux-en-Y gastric bypass. Am. J. Gastroenterol..

[8] Shen Y., Xiao M., Weng J., Yang L., Feng Y., Ye Y., Zheng P. (2023). Diagnosis and treatment of postoperative bleeding in patients after gastrectomy: A retrospective case series study. J. Gastrointest. Oncol..

[9] Wichmann D., Fusco S., Werner C.R., Voesch S., Duckworth-Mothes B., Schweizer U., Stüker D., Königsrainer A., Thiel K., Quante M. (2022). Endoscopic Management for Post-Surgical Complications after Resection of Esophageal Cancer. Cancers.

[10] Heneghan H.M., Meron-Eldar S., Yenumula P., Rogula T., Brethauer S.A., Schauer P.R. (2012). Incidence and management of bleeding complications after gastric bypass surgery in the morbidly obese. Surg. Obes. Relat. Dis. Off. J. Am. Soc. Bariatr. Surg..

[11] Mañas-Gómez M.J., Rodríguez-Revuelto R., Balsells-Valls J., Olsina-Kissler J.J., Caralt-Barba M., Pérez-Lafuente M., Charco-Torra R. (2011). Post-pancreaticoduodenectomy hemorrhage. Incidence, diagnosis, and treatment. World J. Surg..

[12] Yekebas E.F., Wolfram L., Cataldegirmen G., Habermann C.R., Bogoevski D., Koenig A.M., Kaifi J., Schurr P.G., Bubenheim M., Nolte-Ernsting C. (2007). Postpancreatectomy hemorrhage: Diagnosis and treatment: An analysis in 1669 consecutive pancreatic resections. Ann. Surg..

[13] Wei H.-K., Wang S.-E., Shyr Y.-M., Tseng H.-S., Tsai W.-C., Chen T.-H., Su C.-H., Wu C.-W., Lui W.-Y. (2009). Risk factors for post-pancreaticoduodenectomy bleeding and finding an innovative approach to treatment. Dig. Surg..

[14] Bang K.B., Shin H.D. (2018). Endoscopic treatment of surgery or procedure-related gastrointestinal bleeding. Gastrointest. Interv..

[15] Song W., Yuan Y., Peng J., Chen J., Han F., Cai S., Zhan W., He Y. (2014). The delayed massive hemorrhage after gastrectomy in patients with gastric cancer: Characteristics, management opinions and risk factors. Eur. J. Surg. Oncol..

[16] Barola S., Fayad L., Hill C., Magnuson T., Schweitzer M., Singh V., Chen Y.-I., Ngamruengphong S., Khashab M.A., Kalloo A.N. (2018). Endoscopic Management of Recalcitrant Marginal Ulcers by Covering the Ulcer Bed. Obes. Surg..

[17] Barola S., Magnuson T., Schweitzer M., Chen Y.-I., Ngamruengphong S., Khashab M.A., Kumbhari V. (2017). Endoscopic Suturing for Massively Bleeding Marginal Ulcer 10 days Post Roux-en-Y Gastric Bypass. Obes. Surg..

[18] Tang S.J., Rivas H., Tang L., Lara L.F., Sreenarasimhaiah J., Rockey D.C. (2007). Endoscopic hemostasis using endoclip in early gastrointestinal hemorrhage after gastric bypass surgery. Obes. Surg..

[19] Granata A., Ligresti D., Curcio G., Barresi L., Tarantino I., Orlando R., Traina M. (2015). Hemospray rescue treatment of gastroenteric anastomotic bleeding. Endoscopy.

[20] Lee Y.-C., Wang H.-P., Yang C.-S., Yang T.-H., Chen J.-H., Lin C.-C., Tsai C.-Y., Chang L.-Y., Huang S.-P., Wu M.-S. (2002). Endoscopic hemostasis of a bleeding marginal ulcer: Hemoclipping or dual therapy with epinephrine injection and heater probe thermocoagulation. J. Gastroenterol. Hepatol..

[21] Park J.Y., Kim Y.-W., Eom B.W., Yoon H.M., Lee J.H., Ryu K.W., Cho S.J., Lee J.Y., Kim C.G., Choi I.J. (2014). Unique patterns and proper management of postgastrectomy bleeding in patients with gastric cancer. Surgery.

[22] Tontini G.E., Naegel A., Albrecht H., Vieth M., Vecchi M., Neurath M.F., Neumann H. (2013). Successful over-the-scope clip (OTSC) treatment for severe bleeding due to anastomotic dehiscence. Endoscopy.

[23] Sapala J.A., Wood M.H., Sapala M.A., Flake T.M.F. (1998). Marginal ulcer after gastric bypass: A prospective 3-year study of 173 patients. Obes. Surg..

[24] Madan A.K., Kuykendall I.V.S.J., Ternovits C.A., Tichansky D.S. (2005). Mallory-Weiss tear after laparoscopic Roux-en-Y gastric bypass. Surg. Obes. Relat. Dis. Off. J. Am. Soc. Bariatr. Surg..

[25] Amr M.A., Alzghari M.J., Polites S.F., Khasawneh M.A., Morris D.S., Baron T.H., Zielinski M.D. (2014). Endoscopy in the early postoperative setting after primary gastrointestinal anastomosis. J. Gastrointest. Surg. Off. J. Soc. Surg. Aliment. Tract..

[26] Chen J.-F., Xu S.-F., Zhao W., Tian Y.-H., Gong L., Yuan W.-S., Dong J.-H. (2015). Diagnostic and therapeutic strategies to manage post-pancreaticoduodenectomy hemorrhage. World J. Surg..

[27] Gralnek I.M., Stanley A.J., Morris A.J., Camus M., Lau J., Lanas A., Laursen S.B., Radaelli F., Papanikolaou I.S., Gonçalves T.C. (2021). Endoscopic diagnosis and management of nonvariceal upper gastrointestinal hemorrhage (NVUGIH): European Society of Gastrointestinal Endoscopy (ESGE) Guideline—Update 2021. Endoscopy.

[28] Limongelli P., Khorsandi S.E., Pai M., Jackson J.E., Tait P., Tierris J., Habib N.A., Williamson R.C., Jiao L.R. (2008). Management of delayed postoperative hemorrhage after pancreaticoduodenectomy: A meta-analysis. Arch. Surg..

[29] Forrest J.H., Finlayson N.D., Shearman D.J. (1974). Endoscopy in gastrointestinal bleeding. Lancet.

[30] Shin J.S., Chen K.W., Lin X.Z., Lin C.Y., Chang T.T., Yang C.C. (1994). Active, bleeding marginal ulcer of Billroth II gastric resection: A clinical experience of 18 patients. Am. J. Gastroenterol..

[31] Gralnek I.M., Dumonceau J.-M., Kuipers E.J., Lanas A., Sanders D.S., Kurien M., Rotondano G., Hucl T., Dinis-Ribeiro M., Marmo R. (2015). Diagnosis and management of nonvariceal upper gastrointestinal hemorrhage: European Society of Gastrointestinal Endoscopy (ESGE) Guideline. Endoscopy.

[32] Guo S.-B., Gong A.-X., Leng J., Ma J., Ge L.-M. (2009). Application of endoscopic hemoclips for nonvariceal bleeding in the upper gastrointestinal tract. World J. Gastroenterol..

[33] Meier B., Wannhoff A., Denzer U., Stathopoulos P., Schumacher B., Albers D., Hoffmeister A., Feisthammel J., Walter B., Meining A. (2022). Over-the-scope-clips versus standard treatment in high-risk patients with acute non-variceal upper gastrointestinal bleeding: A randomised controlled trial (STING-2). Gut.

[34] Honegger C., Valli P.V., Wiegand N., Bauerfeind P., Gubler C. (2017). Establishment of Over-The-Scope-Clips (OTSC^®^) in daily endoscopic routine. United Eur. Gastroenterol. J..

[35] Barkun A.N., Martel M., Toubouti Y., Rahme E., Bardou M. (2009). Endoscopic hemostasis in peptic ulcer bleeding for patients with high-risk lesions: A series of meta-analyses. Gastrointest. Endosc..

[36] Horák P., Peregrinová M., Erbenová A., Žižková T., Fulík J., Fanta J. (2023). Pneumoperitoneum, pneumomediastinum and subcutaneous emphysema following argon plasma coagulation treatment of colonic angioectasia. Perspect. Surg..

[37] Hajibandeh S., Hajibandeh S., Khan R.M., Malik S., Mansour M., Kausar A., Subar D. (2017). Stapled anastomosis versus hand-sewn anastomosis of gastro/duodenojejunostomy in pancreaticoduodenectomy: A systematic review and meta-analysis. Int. J. Surg..

[38] Vitali F., Naegel A., Atreya R., Zopf S., Neufert C., Siebler J., Neurath M.F., Rath T. (2019). Comparison of Hemospray^®^ and Endoclot^TM^ for the treatment of gastrointestinal bleeding. World J. Gastroenterol..

[39] Branchi F., Klingenberg-Noftz R., Friedrich K., Bürgel N., Daum S., Buchkremer J., Sonnenberg E., Schumann M., Treese C., Tröger H. (2022). PuraStat in gastrointestinal bleeding: Results of a prospective multicentre observational pilot study. Surg. Endosc..

[40] Holster I.L., Kuipers E.J., Tjwa E.T.T.L. (2013). Hemospray in the treatment of upper gastrointestinal hemorrhage in patients on antithrombotic therapy. Endoscopy.

[41] Sung J., Luo D., Wu J., Ching J., Chan F., Lau J., Mack S., Ducharme R., Okolo P., Canto M. (2011). Early clinical experience of the safety and effectiveness of Hemospray in achieving hemostasis in patients with acute peptic ulcer bleeding. Endoscopy.

[42] Holster I.L., van Beusekom H.M.M., Kuipers E.J., Leebeek F.W.G., de Maat M.P.M., Tjwa E.T.T.L. (2015). Effects of a hemostatic powder hemospray on coagulation and clot formation. Endoscopy.

[43] Parsi M.A., Jang S. (2014). Hemospray for diffuse anastomotic bleeding. Gastrointest. Endosc..

[44] Beg S., Al-Bakir I., Bhuva M., Patel J., Fullard M., Leahy A. (2015). Early clinical experience of the safety and efficacy of EndoClot in the management of non-variceal upper gastrointestinal bleeding. Endosc. Int. Open.

[45] Uraoka T., Ochiai Y., Fujimoto A., Goto O., Kawahara Y., Kobayashi N., Kanai T., Matsuda S., Kitagawa Y., Yahagi N. (2016). A novel fully synthetic and self-assembled peptide solution for endoscopic submucosal dissection-induced ulcer in the stomach. Gastrointest. Endosc..

[46] Subramaniam S., Kandiah K., Thayalasekaran S., Longcroft-Wheaton G., Bhandari P. (2019). Haemostasis and prevention of bleeding related to ER: The role of a novel self-assembling peptide. United Eur. Gastroenterol. J..

[47] Pioche M., Camus M., Rivory J., Leblanc S., Lienhart I., Barret M., Chaussade S., Saurin J.-C., Prat F., Ponchon T. (2016). A self-assembling matrix-forming gel can be easily and safely applied to prevent delayed bleeding after endoscopic resections. Endosc. Int. Open.

[48] de Nucci G., Reati R., Arena I., Bezzio C., Devani M., Della Corte C., Morganti D., Mandelli E.D., Omazzi B., Redaelli D. (2020). Efficacy of a novel self-assembling peptide hemostatic gel as rescue therapy for refractory acute gastrointestinal bleeding. Endoscopy.

[49] Kumbhari V., le Roux C.W., Cohen R.V. (2021). Endoscopic Evaluation and Management of Late Complications After Bariatric Surgery: A Narrative Review. Obes. Surg..

[50] Jirapinyo P., Watson R.R., Thompson C.C. (2012). Use of a novel endoscopic suturing device to treat recalcitrant marginal ulceration (with video). Gastrointest. Endosc..

